# Exploring the mechanism of Qingre Yishen Xiaozheng formula in treating diabetic kidney disease via the HIF-1α/HO-1 signaling pathway: an integrated network pharmacology and experimental study

**DOI:** 10.3389/fphar.2025.1661050

**Published:** 2025-09-26

**Authors:** Fei Han, Jieyu Shou, Yaoxian Wang, Zhejing Tian, Huijuan Zheng, Lanying Liu, Weijing Liu

**Affiliations:** ^1^ Beijing University of Chinese Medicine Third Affliated Hospital, Beijing, China; ^2^ Dongzhimen Hospital Affliated to Beijing University of Chinese Medicine, Beijing, China

**Keywords:** Qing-Re-Yi-Shen-Xiao-Zheng formula, diabetic kidney disease, network pharmacology, ferroptosis, HIF-1a/HO-1 signalling pathway

## Abstract

**Ethnopharmacological relevance:**

The Qingre Yishen Xiaozheng Formula (QRYSXZF) is a traditional Chinese medicine prescription developed based on the “clearing heat and resolving stasis” principle, clinically applied for the treatment of diabetic kidney disease (DKD).

**Aim of the study:**

To investigate the therapeutic effects of QRYSXZF on DKD and elucidate its underlying mechanisms through integrated network pharmacology and experimental validation, focusing on the HIF-1α/HO-1 signaling pathway and ferroptosis regulation.

**Materials and methods:**

Active components of QRYSXZF were screened using the TCMSP database (OB ≥ 30%, DL ≥ 0.18), and a herb-compound-target network was constructed via Cytoscape 3.8.0. DKD-related targets were retrieved from GeneCards, OMIM, and TTD databases. Protein-protein interaction (PPI) networks, GO/KEGG enrichment analyses, and molecular docking (PyMOL/AutoDock) were performed to predict core targets and pathways. *In vivo*, a DKD rat model was established by unilateral nephrectomy combined with streptozotocin (STZ) injection, followed by 12-week QRYSXZF treatment. Renal function markers (BUN, 24h-UTP, KIM-1, NGAL), oxidative stress (SOD, MDA, GSH-Px), iron metabolism (SI, SF, TF), and ferroptosis-related proteins (GPX4, ACSL4, FTH1, NCOA4) were analyzed. Histopathological changes were assessed by H&E, PAS, and Masson staining, while HIF-1α/HO-1 pathway activity was evaluated via Western blot.

**Results:**

Network pharmacology identified 153 shared targets between QRYSXZF and DKD, with quercetin, kaempferol, and β-sitosterol as core active components, while KEGG analysis highlighted the HIF-1 signaling pathway as a key mechanism. In DKD rats, QRYSXZF significantly improved renal function by reducing BUN, Cys-C, KIM-1 and NGAL, attenuated oxidative stress through increasing SOD/GSH-Px and decreasing MDA, regulated iron metabolism by lowering SF and elevating TF, suppressed ferroptosis via upregulating GPX4/FTH1 and downregulating ACSL4/NCOA4, and inhibited HIF-1α/HO-1 pathway activation, with molecular docking confirming stable binding between QRYSXZF components and HIF1A/HMOX1.

**Conclusion:**

QRYSXZF alleviates DKD progression by modulating the HIF-1α/HO-1 pathway to reduce ferroptosis, oxidative stress, and iron overload, providing a scientific basis for its clinical application in DKD management.

## 1 Introduction

Diabetic Kidney Disease (DKD), as one of the major microvascular complications of diabetes, has become a significant contributor to end-stage renal disease (Zhang et al., 2020a). With the global prevalence of DKD continuing to rise, its burden on public health systems is becoming increasingly prominent. Ferroptosis, a novel form of regulated cell death characterized by iron dependence and lipid peroxidation, has been implicated in various diseases, including DKD (Zhang et al., 2021). Unlike traditional cell death pathways such as apoptosis and necrosis, ferroptosis exhibits distinct morphological features, including mitochondrial shrinkage, increased membrane density, and reduced cristae (Wang et al., 2022). Studies indicate that ferroptosis plays a critical role in DKD pathogenesis, as a high-glucose microenvironment promotes iron accumulation and lipid peroxidation in renal tubular epithelial cells, triggering ferroptosis and subsequent tubular injury (Zhang et al., 2022a). Notably, ferroptosis not only directly damages tubular epithelial cells but also exacerbates renal interstitial fibrosis and functional decline by releasing inflammatory mediators (Zhang et al., 2022b). The interplay between ferroptosis, oxidative stress, inflammation, and mitochondrial dysfunction collectively drives DKD progression (Yan et al., 2021). Oxidative stress, a central pathological mechanism in DKD, leads to excessive ROS production under hyperglycemic conditions due to mitochondrial dysfunction and NADPH oxidase activation, further aggravating lipid peroxidation and ferroptosis (Zhang et al., 2020b). Thus, ferroptosis is not merely a pathological manifestation but a key driver of DKD progression. Reports suggest that hypoxia-inducible factor-1α (HIF-1α) activation promotes ferroptosis; under hypoxic conditions, inhibited hydroxylation of HIF-1α allows its dimerization with HIF-1β and nuclear translocation, upregulating downstream targets such as heme oxygenase-1 (HO-1), thereby exacerbating ROS generation, lipid peroxidation, and iron overload in renal tubules, ultimately inducing ferroptosis and aggravating kidney injury (Gao et al., 2020).

In recent years, integrated traditional Chinese and Western medicine strategies for DKD have gained attention, with traditional Chinese medicine (TCM) demonstrating unique advantages in alleviating clinical symptoms and delaying disease progression. Based on extensive clinical experience, Professor Wang Yaoxian proposed that the core pathogenesis of DKD involves “internal heat-induced stasis” (Wang et al., 2020a) and established the therapeutic principle of “clearing heat and resolving stasis.” Preliminary studies confirmed that this approach significantly benefits early-to-mid-stage DKD, potentially delaying progression by modulating ferroptosis, regulating iron metabolism-related proteins, and reducing intracellular iron accumulation (Feng et al., 2021). In addition, our team has demonstrated through a pilot clinical trial that QRYSXZF can effectively improve clinical symptoms in DKD patients, reduce renal function impairment, enhance glucose metabolism, and alleviate iron overload, thereby potentially influencing ferroptosis and delaying the progression of DKD. However, the core pharmacological targets of this treatment, particularly whether it acts on renal tubules via HIF-1α, remain unclear. Guided by the principle of clearing heat and resolving stasis, the Qingre Yishen Xiaozheng Formula (QRYSXZF) was formulated, leading to the hypothesis that “QRYSXZF may inhibit ferroptosis and delay DKD progression by regulating the HIF-1α/HO-1 pathway.”

To further elucidate the mechanism of QRYSXZF in DKD treatment, this study integrates network pharmacology and experimental validation to investigate whether QRYSXZF exerts antioxidative, anti-ferroptotic, and renoprotective effects via the HIF-1α/HO-1 pathway. The findings aim to clarify the scientific basis for QRYSXZF in DKD management, provide a stronger theoretical foundation for its clinical application, and explore novel strategies for TCM-based DKD therapy.

## 2 Materials and methods

### 2.1 Network pharmacology

This study employed the constituent herbs of QRYSXZF as key terms to screen active compounds through the TCMSP database, applying selection criteria of oral bioavailability (OB) ≥ 30% and drug-likeness (DL) ≥ 0.18. Unrecorded compounds were supplemented via literature review and HERB database. After standardization using UniProt database, a comprehensive “herb-compound-target” database was established. Concurrently, DKD-related targets were retrieved from GeneCards, OMIM, and TTD databases, followed by integration and deduplication. The drug-disease common targets were identified using Venny 2.1 to generate a Venn diagram. A herb-compound-target interaction network was constructed using Cytoscape 3.8.0, with node degree values serving as indicators to predict core active components. The common targets were imported into STRING database (https://cn.string-db.org/) to construct a protein-protein interaction (PPI) network. Core targets were subsequently filtered using Cytoscape’s CentiScaPe 2.2 plugin based on three topological parameters: Betweenness, Closeness, and Degree. Gene Ontology (GO) functional enrichment analysis (including Cellular Components [CC], Biological Processes [BP], and Molecular Functions [MF]) and KEGG pathway enrichment were performed using Metascape (https://www.metascape.org). Significant enrichment results were visualized through the Microbioinformatics platform.

### 2.2 Molecular docking

This study employed Cytoscape to screen core active compounds and their targets from QRYSXZF. Protein structures were retrieved from the PDB database, while small-molecule files were obtained from TCMSP. Protein structures were preprocessed using PyMOL (removing water molecules and optimizing structures), followed by hydrogenation and parameter configuration in AutoDock. Semi-flexible molecular docking was then performed. Finally, binding energies were analyzed, and docking results were visualized using PyMOL.

### 2.3 Experimental animals

This study utilized SPF-grade male SD rats (6–8 weeks old, weighing 200 ± 20 g), supplied by Beijing Vital River Laboratory Animal Technology Co., Ltd. The animals were housed in a barrier environment (24 °C ± 2 °C, 60% ± 5% humidity, 12-h light/dark cycle) with free access to standard chow and sterilized water. All experimental procedures complied with the Animal Care Guidelines of Beijing University of Chinese Medicine and were approved by the Institutional Animal Ethics Committee (NO. DZMYY24-19).

### 2.4 Establishment of the DKD model

The DKD rat model was established through unilateral nephrectomy combined with streptozotocin (STZ) injection (55 mg/kg,Dissolved in citric acid buffer of Citrus limon, pH 4.5). After successful model induction, animals were randomized based on blood glucose levels. Following exclusion of 2 unsuccessful model rats and 8 deceased animals, the remaining successfully modeled rats were equally divided into three groups (n = 10 per group): model group, QRYSXZF group, and dapagliflozin group. Oral gavage interventions began the day after successful modeling and continued for 12 weeks, with dosages administered according to the Pharmacological Experimental Methods. The control group received 3 mL saline per rat daily, while the model group was given 3 mL saline per rat daily. The QRYSXZF group was administered 11.34 g/kg/day of QRYSXZF, and the dapagliflozin group received 0.9 mg/kg/day of dapagliflozin dissolved in saline. At the experimental endpoint, rats were anesthetized for abdominal aortic blood collection and kidney tissue extraction. Renal tissues were weighed and the cortex was longitudinally divided into four portions: one fixed in 4% paraformaldehyde for paraffin and OCT embedding, one preserved in 2.5% glutaraldehyde for electron microscopy, and the remaining two snap-frozen in liquid nitrogen for subsequent Western blot analysis.

### 2.5 Biochemical analyses of blood, urine and renal tissues

The 24-h urinary total protein (24h-UTP), serum urea nitrogen (BUN), serum creatinine (Scr), serum cystatin C (Cys-C), serum transferrin (TF), and serum iron (SI) were analyzed by the Clinical Laboratory of Dongzhimen Hospital, Beijing University of Chinese Medicine. Serum ferritin (SF) were determined using commercial kits (Jianglai Biology, Shanghai, China). Renal tissue glutathione (GSH), malondialdehyde (MDA), and superoxide dismutase (SOD) were measured with assay kits from Nanjing Jiancheng Bioengineering Institute (Nanjing, China), while kidney injury molecule-1 (KIM-1) and neutrophil gelatinase-associated lipocalin (NGAL) levels were quantified using ELISA kits (Jianglai Biology, Shanghai, China).

### 2.6 Renal morphological analyses and histological staining

The renal tissues were observed after hematoxylin-eosin (H&E), Periodic acid-Schiff (PAS) and Masson staining, while the transmission electron microscopy samples were examined after glutaraldehyde-osmium fixation, dehydration, embedding, and double-staining of ultrathin sections.

### 2.7 Immunohistochemistry

Antigen retrieval was performed in citrate buffer (pH 6.0) at 95 °C for 20 min after deparaffinization. Endogenous peroxidase was blocked with 3% H_2_O_2_ (RT, 20 min). Primary antibody incubation proceeded at 4 °C overnight, followed by 1-h rewarming. After 20-min amplification at 37 °C, secondary antibody incubation was conducted (37 °C, 20 min). DAB development and hematoxylin counterstaining preceded standard mounting procedures.

### 2.8 Western blotting analysis

Kidney tissues were rinsed with PBS and homogenized in RIPA lysis buffer containing protease inhibitors under ice-cold conditions. After centrifugation at 12,000 rpm for 15 min, the protein supernatant was collected for quantification using the BCA method. SDS-PAGE gels (including separating and stacking gels) were prepared, with 5 μg protein loaded per lane. Electrophoresis was performed at 60–100 V until the bromophenol blue tracking dye migrated to the bottom. Protein transfer was conducted using wet transfer method (200 mA, 2 h, ice bath). Membranes were incubated with the following primary antibodies at 4 °C overnight: HO-1/HMOX1 (1:1,000, Proteintech, Wuhan, China), ACSL4/FACL4 (1:1,000, Proteintech, Wuhan, China), HIF-1a (1:1,000, Abcam, Cambridge, MA,United States of America), GPX 4 (1:1,000, Abcam, Cambridge, MA,United States of America),NCOA4 (1:1,000, Abcam, Cambridge, MA,United States of America), FTH1(1:1,000, Cell Signaling, Danvers, MA, United States of America), Caspase-3 (1:1,000, Cell Signaling, Danvers, MA, United States of America),TGF-β(1:1,000, Cell Signaling, Danvers, MA, United States of America) and β-actin.

(1:1,000, Proteintech, Wuhan, China), followed by 1-h room temperature incubation with secondary antibodies (1:1,000, LamAbiotech, Wuhan, China). After thorough washing with TBST buffer, protein bands were visualized by chemiluminescence detection.

### 2.9 Statistical analysis

All statistical analyses were performed using GraphPad Prism 9.0.0 for data processing and graph generation. Quantitative analysis of Western blot band intensities was conducted using ImageJ. Experimental data are presented as mean ± SEM (standard error of the mean). For normally distributed data, one-way ANOVA was employed for intergroup comparisons. Non-normally distributed data were analyzed using nonparametric tests. The significance level was set at *α* = 0.05, with *P* < 0.05 considered statistically significant.

## 3 Results

### 3.1 Network pharmacology investigation of the mechanism by which QRYXSZF treats DKD

**FIGURE 1:**
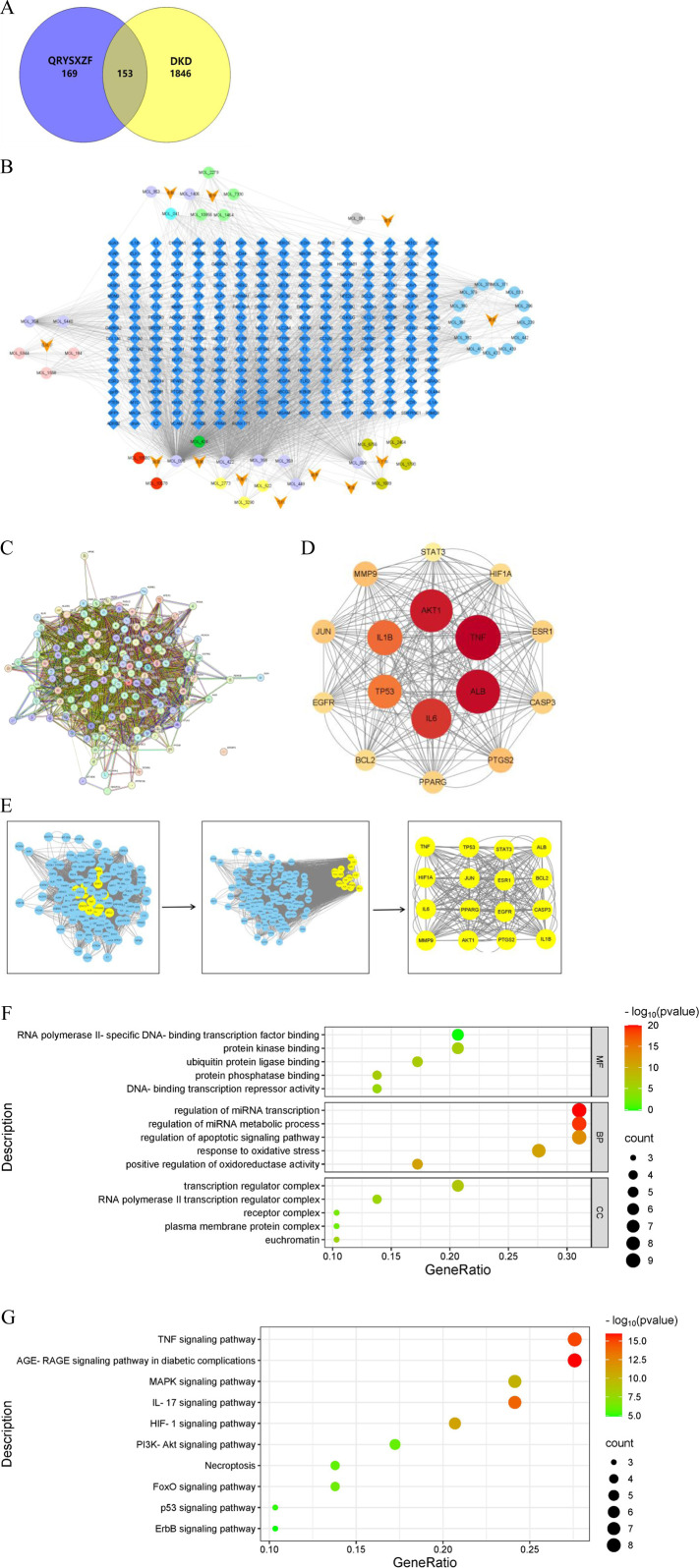
**(A)** Venn diagram of DKD and QRYXSZF targets. **(B)** QRYXSZF-Compound-Target Network Diagram. **(C)** Protein-protein interaction (PPI) network. **(D)** Core Target Interaction Network. **(E)** Key Gene Screening Flowchart. **(F)** GO enrichment analysis. **(G)** KEGG pathway enrichment analysis.

Through systematic screening and deduplication of bioactive compounds derived from traditional Chinese medicine, we identified 153 shared targets between the herbal components and the disease pathology. Network pharmacology analysis pinpointed five core bioactive constituents: quercetin, kaempferol, β-sitosterol, luteolin, and isorhamnetin. The constructed protein-protein interaction (PPI) network further elucidated 16 pivotal targets, namely, TNF, ALB, AKT1, IL6, IL1β, TP53, MMP9, PTGS2, JUN, PPARG, CASP3, ESR1, BCL2, EGFR, HIF1A, and STAT3. GO functional enrichment analysis demonstrated that the therapeutic mechanisms may principally involve specific DNA-binding transcription factor interactions, modulation of protein kinase activity, regulation of apoptotic signaling pathways, and mediation of oxidative stress responses. KEGG pathway analysis revealed the potential involvement of multiple signaling cascades, including AGE-RAGE, TNF, IL-17, HIF-1, MAPK, FoxO, PI3K-AKT, necroptosis, p53, and ErbB pathways. These findings collectively suggest a multi-component, multi-target therapeutic paradigm characteristic of traditional Chinese medicine interventions (Figure 1).

### 3.2 Molecular docking shows the ability of QRYXSZF to bind its core targets

**FIGURE 2:**
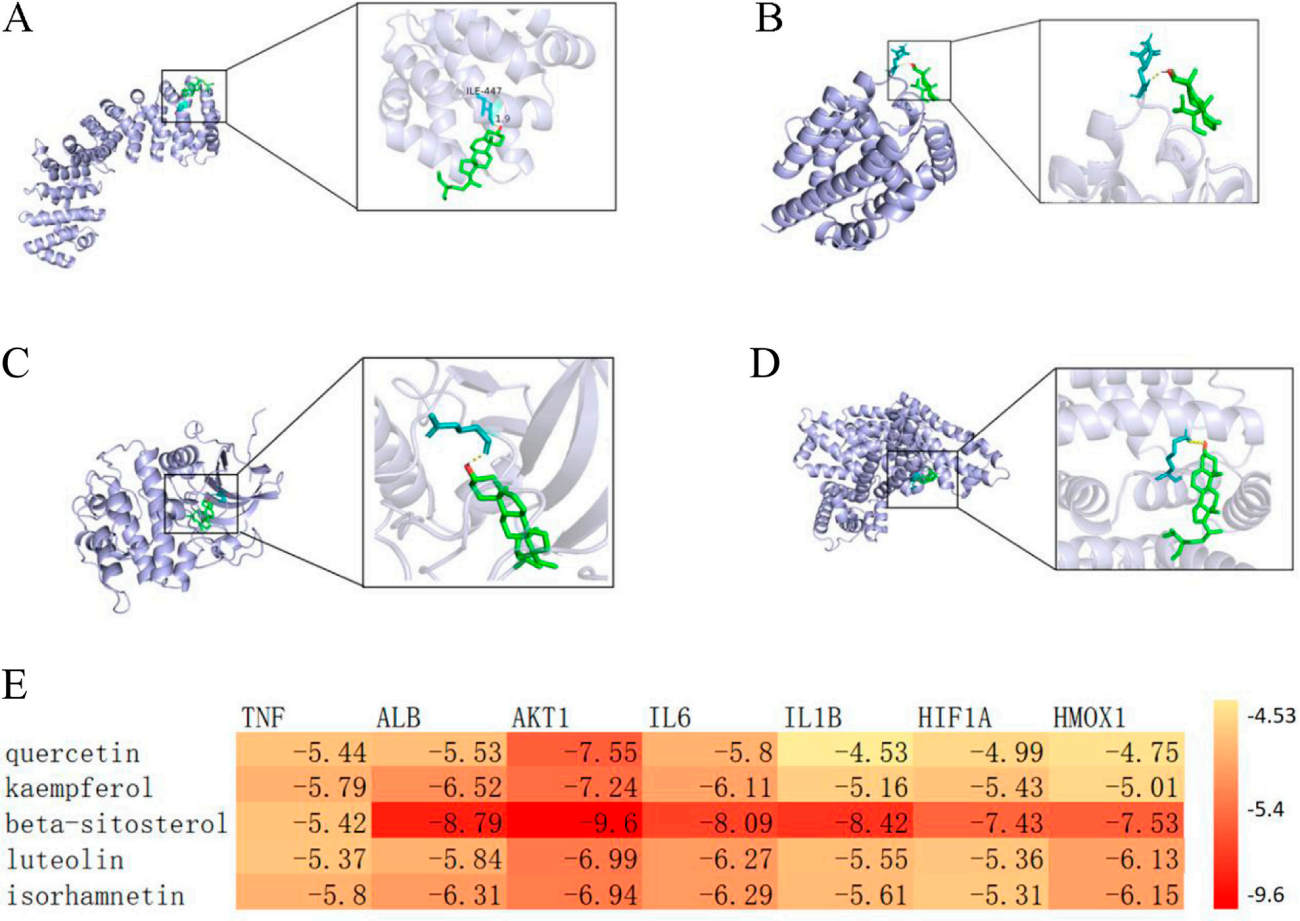
**(A)** Molecular docking visualization of β-sitosterol with HIF1A. **(B)** Molecular docking visualization of β-sitosterol with HMOX1. **(C)** Molecular docking visualization of β-sitosterol with AKT1. **(D)** Molecular docking visualization of β-sitosterol with ALB. **(E)** Heatmap of molecular docking between key targets and core bioactive compounds from traditional Chinese medicine.

The combined results of enrichment analysis and Cytoscape network construction, supported by literature review, indicate that the therapeutic mechanisms primarily involve modulation of inflammatory responses, oxidative stress, cellular autophagy/apoptosis, and ferroptosis. Based on network topology analysis, we focused on the top five targets by degree value (TNF, ALB, AKT1, IL6, and IL1B) along with ferroptosis-related targets HIF1A and HMOX1. The five highest-ranking bioactive compounds (quercetin, kaempferol, β-sitosterol, luteolin, and isorhamnetin) were selected for molecular docking with these targets. The docking results, visualized as a heatmap with representative binding poses shown in Figure 2, demonstrated binding affinities < -4.25 kcal/mol for all compound-target pairs, with stable hydrogen bond formation observed. These findings validate the network pharmacology predictions, showing significant binding potential between the identified core compounds and key therapeutic targets across multiple pathological pathways.

### 3.3 QRYSXZF ameliorates general physiological conditions in DKD rats

**FIGURE 3:**
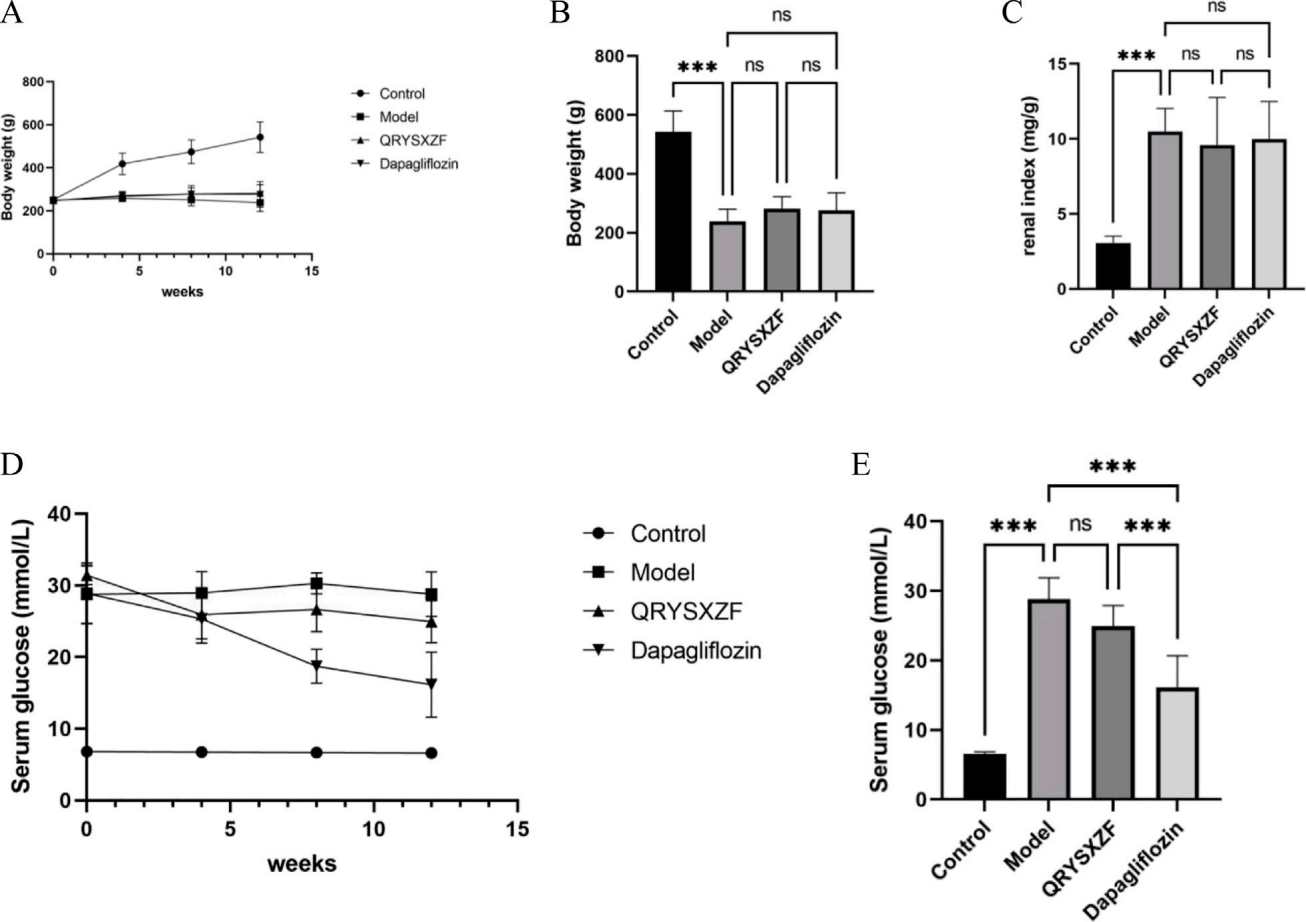
**(A)** The body weight change trends of rats in each group from 0 to 12 weeks were recorded with every 4 weeks serving as a marked time point. **(B)** The comparison of body weight among the different rat groups after the 12-week intervention period. **(C)** The comparison of kidney-to-body weight ratio among the experimental rat groups after the 12-week intervention period, where kidney-to-body weight ratio was calculated as kidney weight (mg) divided by body weight (G). **(D)** The trends of random blood glucose levels in each rat group from 0 to 12 weeks were monitored with measurements recorded at 4-week intervals as designated time points. **(E)** The comparison of random blood glucose levels among experimental rat groups after the 12-week intervention period. *P < 0.05,**P < 0.01,***P < 0.001.

During the experimental period, behavioral observations revealed distinct physiological states among groups: control rats maintained normal healthy conditions, while model group animals exhibited marked pathological manifestations including lethargy, reduced activity, sluggish responses, emaciation, yellowish fur discoloration, and classic polydipsia-polyphagia-polyuria triad. Both QRYSXZF and dapagliflozin interventions improved mental status and mobility with partial fur color recovery to pale yellow, though food/water intake and urinary output remained comparable to model group. As shown in Figure 3A, control rats demonstrated progressive weight gain throughout the study, whereas model animals showed slight weight reduction with minimal growth in treatment groups. After 12-week interventions, all experimental groups exhibited significantly lower body weights versus controls (P < 0.05), most pronounced in model group (Figure 3B). Kidney-to-body weight ratios were substantially elevated in all treatment groups compared to controls (Figure 3C). Glycemic monitoring (Figure 3D) demonstrated dramatic glucose reduction in dapagliflozin group versus modest decline with QRYSXZF after 12 weeks, while model group maintained hyperglycemia. Longitudinal analysis (Figure 3E) confirmed stable normoglycemia in controls versus persistent hyperglycemia in model group (P > 0.05). Dapagliflozin achieved significant glucose-lowering versus model (P < 0.001), whereas QRYSXZF showed no statistically significant hypoglycemic effect.

### 3.4 QRYSXZF improves renal function and attenuates kidney injury in DKD rats

**FIGURE 4:**
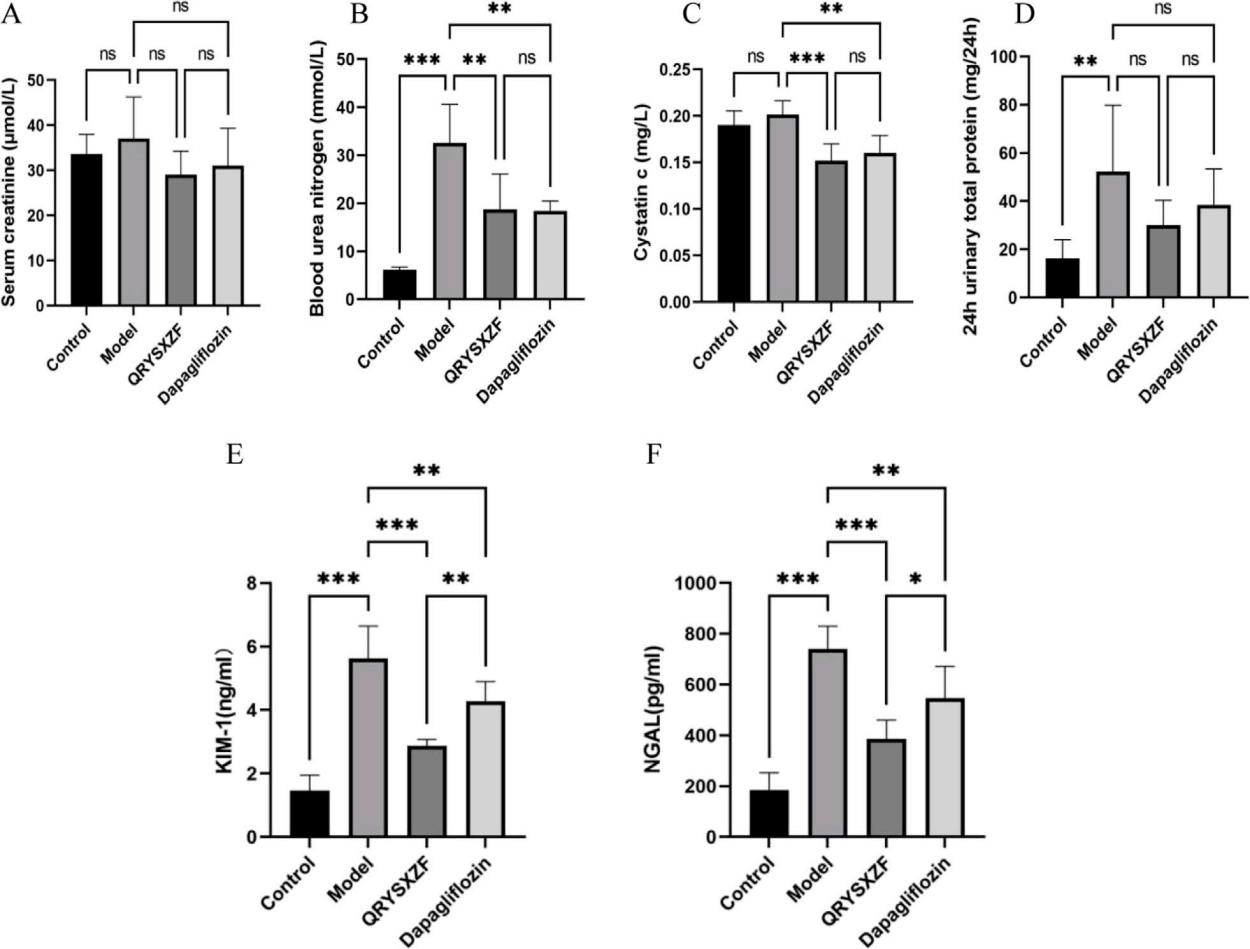
Comparative analysis of renal function and renal injury status among the experimental rat groups after 12 weeks of intervention (n = 6). **(A)** Serum creatinine. **(B)** Blood urea nitrogen. **(C)** Cystatin C. **(D)** 24-h urinary protein. **(E)** Kidney injury molecule 1. **(F)** Neutrophil gelatinase-associated lipocalin. *P < 0.05,**P < 0.01,***P < 0.001.

Comparative analysis of renal function parameters after 12-week drug interventions (Figures 4A–D) revealed no significant differences in Scr levels among all four groups (P > 0.05). The model group exhibited significantly elevated BUN and 24h-UTP compared to controls (both P < 0.01), while both QRYSXZF and dapagliflozin treatment groups showed markedly reduced BUN and Cys-C levels versus model animals (P < 0.01), with 24h-UTP demonstrating a decreasing trend that did not reach statistical significance (P > 0.05). Evaluation of renal injury biomarkers KIM-1 and NGAL (Figures 4E,F) demonstrated low baseline expression in controls indicating intact renal function, whereas model group displayed significantly upregulated levels (P < 0.001), confirming substantial kidney damage. Both QRYSXZF (P < 0.001) and dapagliflozin (P < 0.01) treatments significantly attenuated KIM-1 and NGAL expression compared to model group, demonstrating renal protective effects.

### 3.5 QRYSXZF ameliorates renal pathological damage and attenuates fibrosis in DKD rats

**FIGURE 5:**
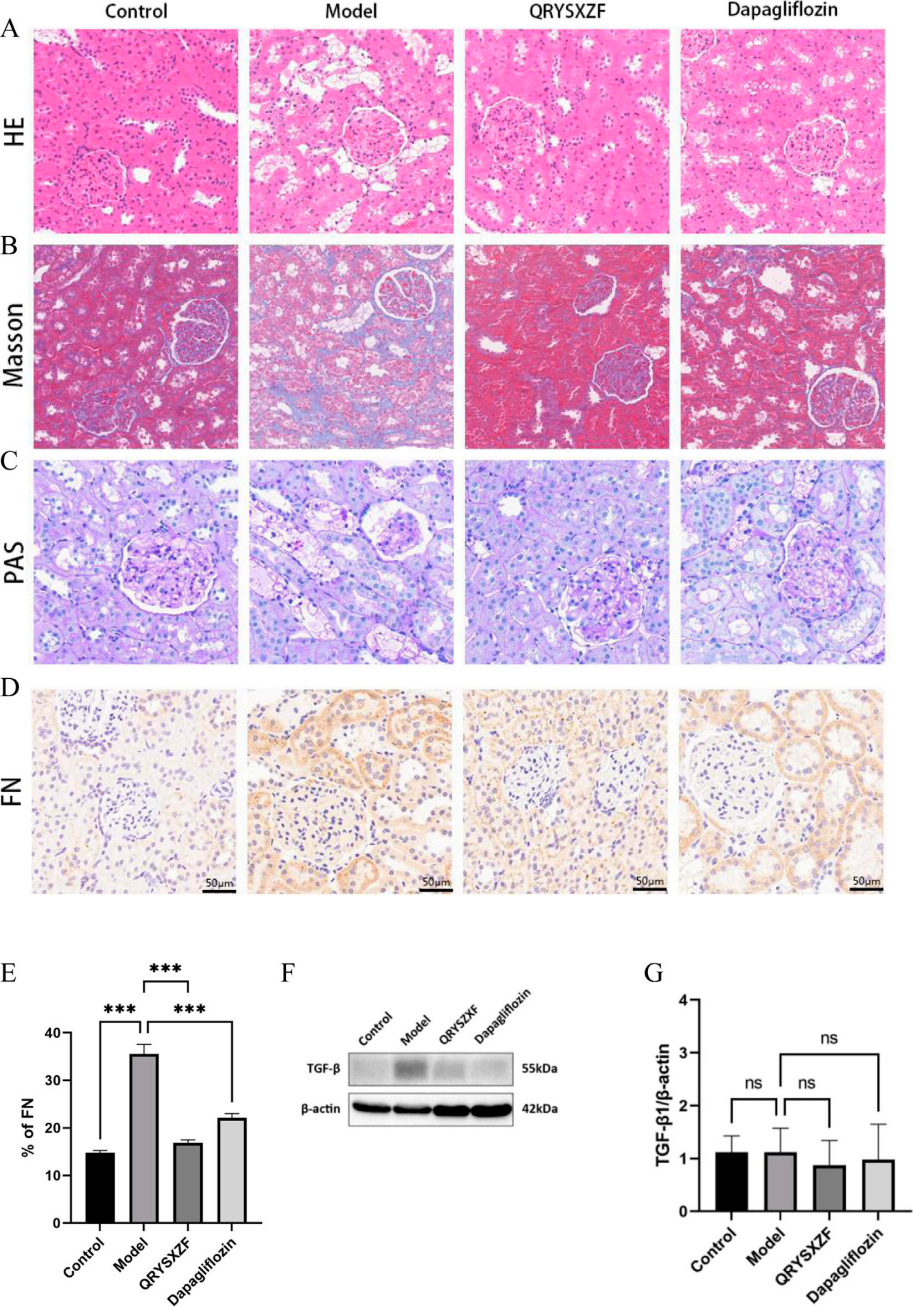
Comparative analysis of renal pathological damage and attenuates fibrosis among the experimental rat groups after 12 weeks of intervention. **(A)** H&E staining. **(B)** Masson’s trichrome. **(C)** PAS staining. **(D)** Immunohistochemistry staining of FN. **(E)** Quantitative analysis of FN immunohistochemical staining. **(F)** Western blot analysis of TGF-β protein expression in renal tissues across experimental rat groups. **(G)** The relative expression level of TGF-β protein. *P < 0.05,**P < 0.01,***P < 0.001.

Histological examination (Figures 5A–C) revealed well-preserved renal tubular architecture with regularly arranged epithelial cells in control groups by H&E staining, while Masson’s trichrome showed minimal collagen deposition and PAS staining demonstrated uniform basement membranes without thickening, confirming normal renal histology. The model group exhibited significant pathological alterations: H&E staining displayed tubular epithelial cell swelling, necrotic detachment and inflammatory infiltration; Masson’s trichrome indicated aggravated interstitial fibrosis with increased collagen accumulation; PAS staining revealed basement membrane thickening and enhanced glycogen deposition. QRYSXZF treatment group demonstrated marked histological improvement: H&E showed attenuated epithelial cell swelling and inflammatory infiltration; Masson’s trichrome exhibited reduced fibrotic areas; PAS staining confirmed ameliorated basement membrane thickening. The dapagliflozin group showed moderate improvement, though less pronounced than QRYSXZF: H&E still presented partial tubular dilation and residual inflammation; Masson’s trichrome displayed decreased collagen fibers; PAS staining indicated mitigated basement membrane thickening. Immunohistochemistry (Figure 5D) demonstrated weak FN protein expression predominantly localized to glomerular basement membranes and peritubular areas in controls, whereas model kidneys showed significantly intensified FN expression extensively distributed across glomeruli, tubules and interstitium. Both QRYSXZF and dapagliflozin groups exhibited substantially reduced FN expression primarily confined to glomerular and peritubular regions with weakened intensity. Western blot analysis (Figures 5E,F) indicated marginally decreased TGF-β expression in QRYSXZF-treated kidneys versus model group, though no statistically significant differences in renal TGF-β levels were observed among all experimental groups.

### 3.6 QRYSXZF ameliorates mitochondrial dysfunction and attenuates renal cellular apoptosis in DKD rats

**FIGURE 6:**
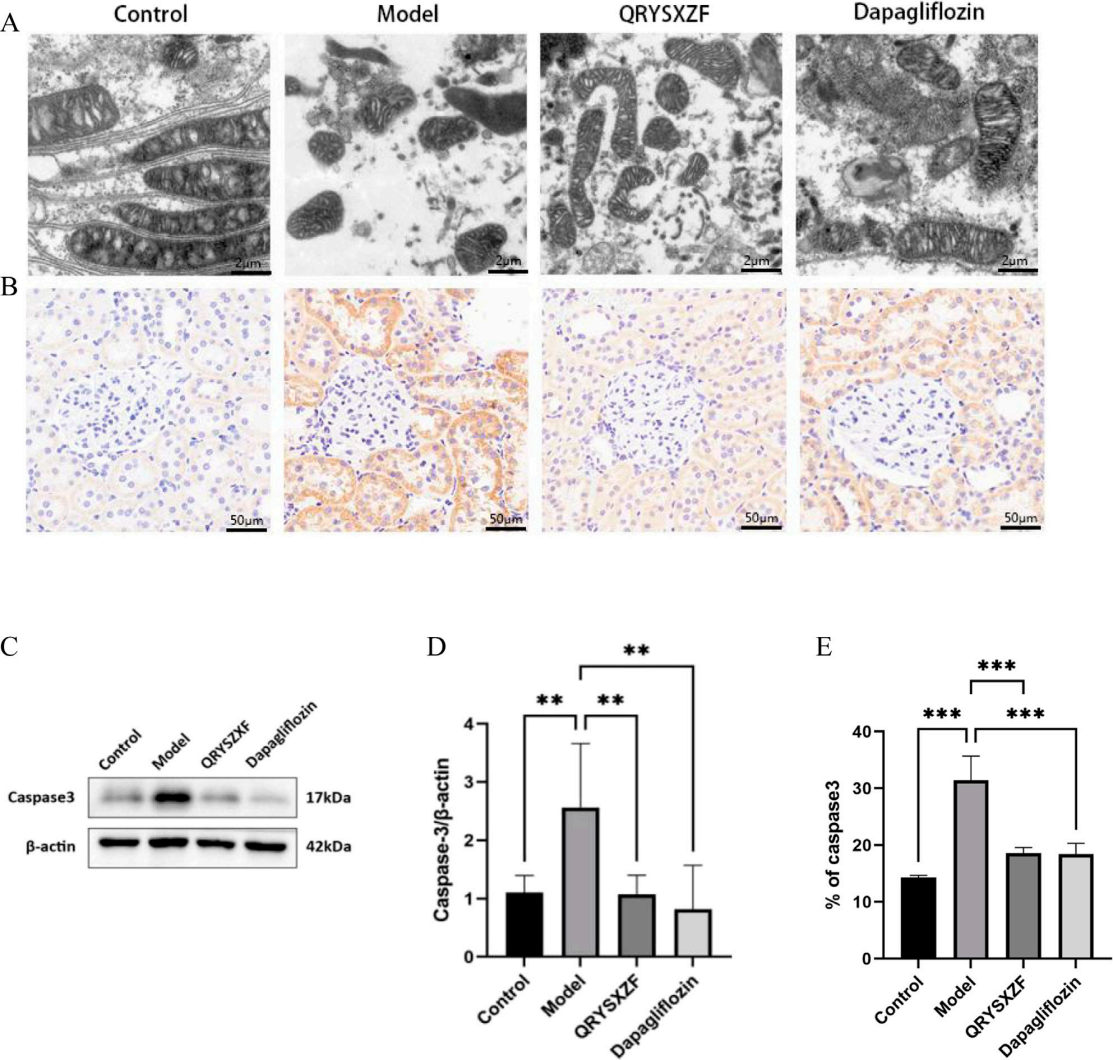
Comparative analysis of mitochondrial dysfunction and renal cellular apoptosis among the experimental rat groups after 12 weeks of intervention (n = 6). **(A)** Comparative assessment of mitochondrial damage across experimental rat groups. **(B)** Immunohistochemistry staining of Caspase-3. **(C)** Western blot analysis of Caspase-3 protein expression in renal tissues across experimental rat groups. **(D)** The relative expression level of Caspase-3 protein. *P < 0.05,**P < 0.01,***P < 0.001. **(E)** Quantitative analysis of Caspase-3 immunohistochemical staining.

Transmission electron microscopy revealed intact mitochondrial structure with clear inner membranes and well-organized cristae in renal cells of control group rats, indicating normal functionality. The model group exhibited significant mitochondrial damage characterized by swelling, cristae fragmentation, vacuolization, and membrane rupture. Both QRYSXZF and dapagliflozin treatment groups demonstrated markedly attenuated mitochondrial damage compared to the model group, showing reduced swelling and cristae disruption with more preserved ultrastructure, suggesting improved mitochondrial morphology and function in both intervention groups (Figure 6A). Immunohistochemical staining (Figures 6B,E) showed elevated Caspase-3 expression in model group kidneys versus controls. QRYSXZF treatment significantly reduced Caspase-3 protein expression, primarily localized in tubular epithelial cells and glomerular areas with markedly decreased intensity. Western blot results (Figures 6C,D) demonstrated low basal levels of Caspase-3 protein expression in control kidneys. Model group kidneys showed significantly upregulated Caspase-3 protein expression compared to controls (P < 0.01). Western blot analysis revealed significantly decreased Caspase-3 protein levels in both QRYSXZF and dapagliflozin groups versus model (P < 0.01).

### 3.7 QRYSXZF ameliorates oxidative stress in DKD rats

**FIGURE 7:**
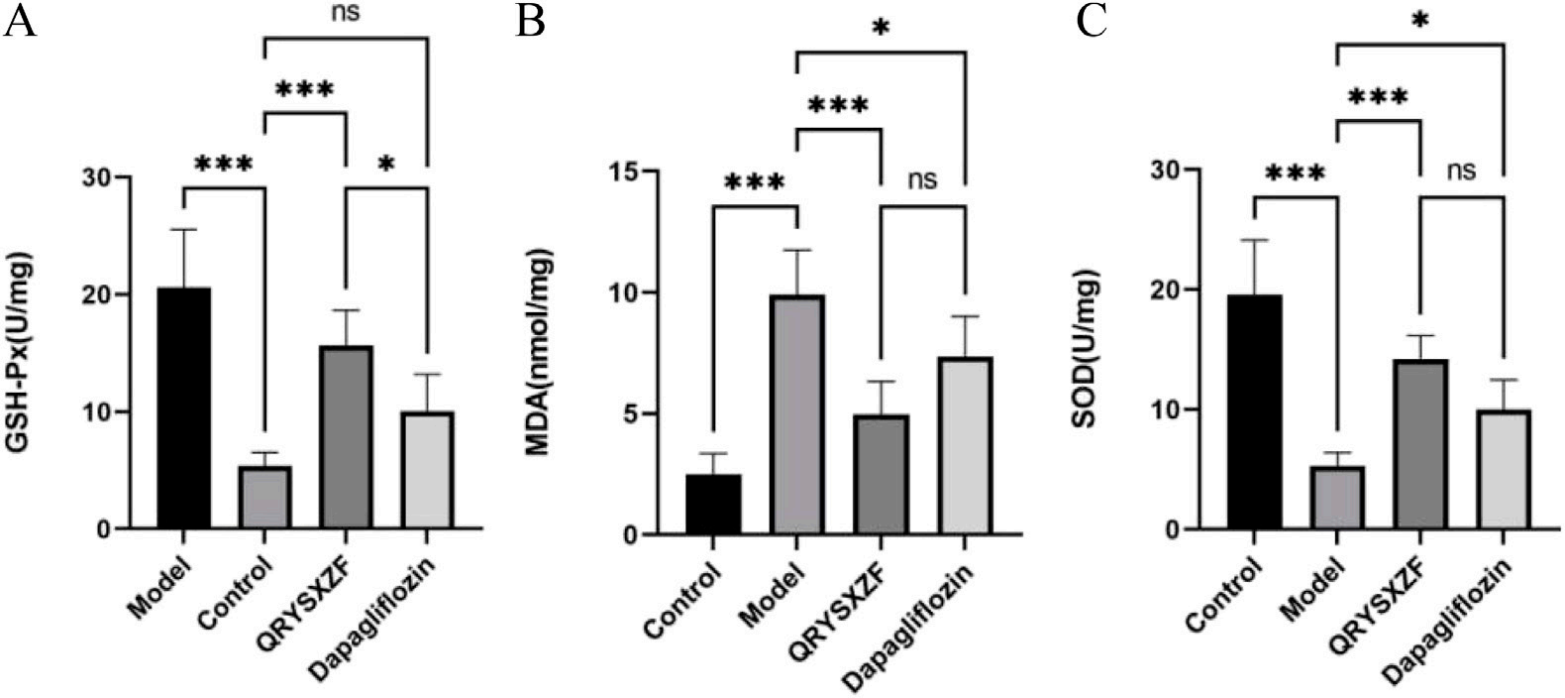
Comparative analysis of oxidative stress markers in renal tissues among experimental rat groups (n = 6). **(A)** Glutathione peroxidase (GSH-Px). **(B)** Malondialdehyde (MDA). **(C)** Superoxide dismutase (SOD). *P < 0.05,**P < 0.01,***P < 0.001.

Analysis of oxidative stress markers (Figure 7) demonstrated significantly decreased renal SOD and GSH-Px levels alongside elevated MDA content in model group rats compared to controls. QRYSXZF treatment markedly increased GSH-Px and SOD activities (both P < 0.001) while reducing MDA levels (P < 0.001) versus model animals. Dapagliflozin administration showed moderate antioxidant effects with statistically significant elevation in SOD (P < 0.05) and reduction in MDA (P < 0.05), though its GSH-Px enhancement did not reach statistical significance, indicating differential regulation of oxidative stress pathways between the two therapeutic interventions.

### 3.8 QRYSXZF alleviates renal iron overload in DKD rats

**FIGURE 8:**
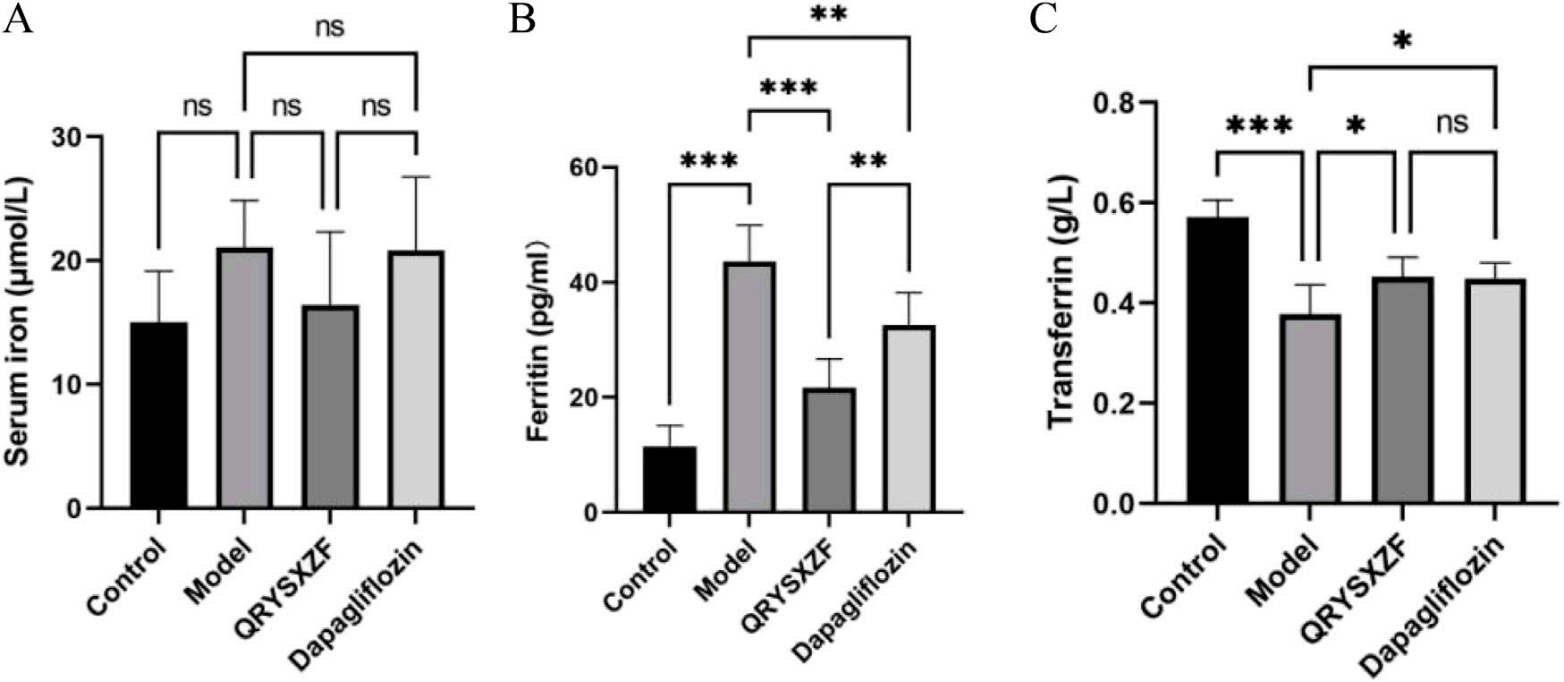
Comparative analysis of iron-related parameters in renal tissues across experimental rat groups (n = 6). **(A)** Serum iron (SI). **(B)** Serum ferritin (SF). **(C)** Transferrin (TF). *P < 0.05,**P < 0.01,***P < 0.001.

The comparative analysis of iron metabolism parameters (Figure 8) showed that the model group exhibited an upward trend in serum iron (SI) levels (P = 0.07), though this did not reach statistical significance. In contrast, serum ferritin (SF) was significantly elevated (P < 0.001), while transferrin (TF) was significantly reduced (P < 0.001). While QRYSXZF treatment showed a non-significant reduction trend in SI levels, it dramatically lowered SF content (P < 0.001) and moderately increased TF levels (P < 0.05) compared to model animals. Dapagliflozin similarly demonstrated significant SF reduction (P < 0.01) and TF elevation (P < 0.05), though with less pronounced effects than QRYSXZF, suggesting both treatments can ameliorate renal iron overload through differential regulation of iron storage and transport mechanisms.

### 3.9 QRYSXZF demonstrated significant regulatory effects on ferroptosis-related markers in DKD rats

**FIGURE 9:**
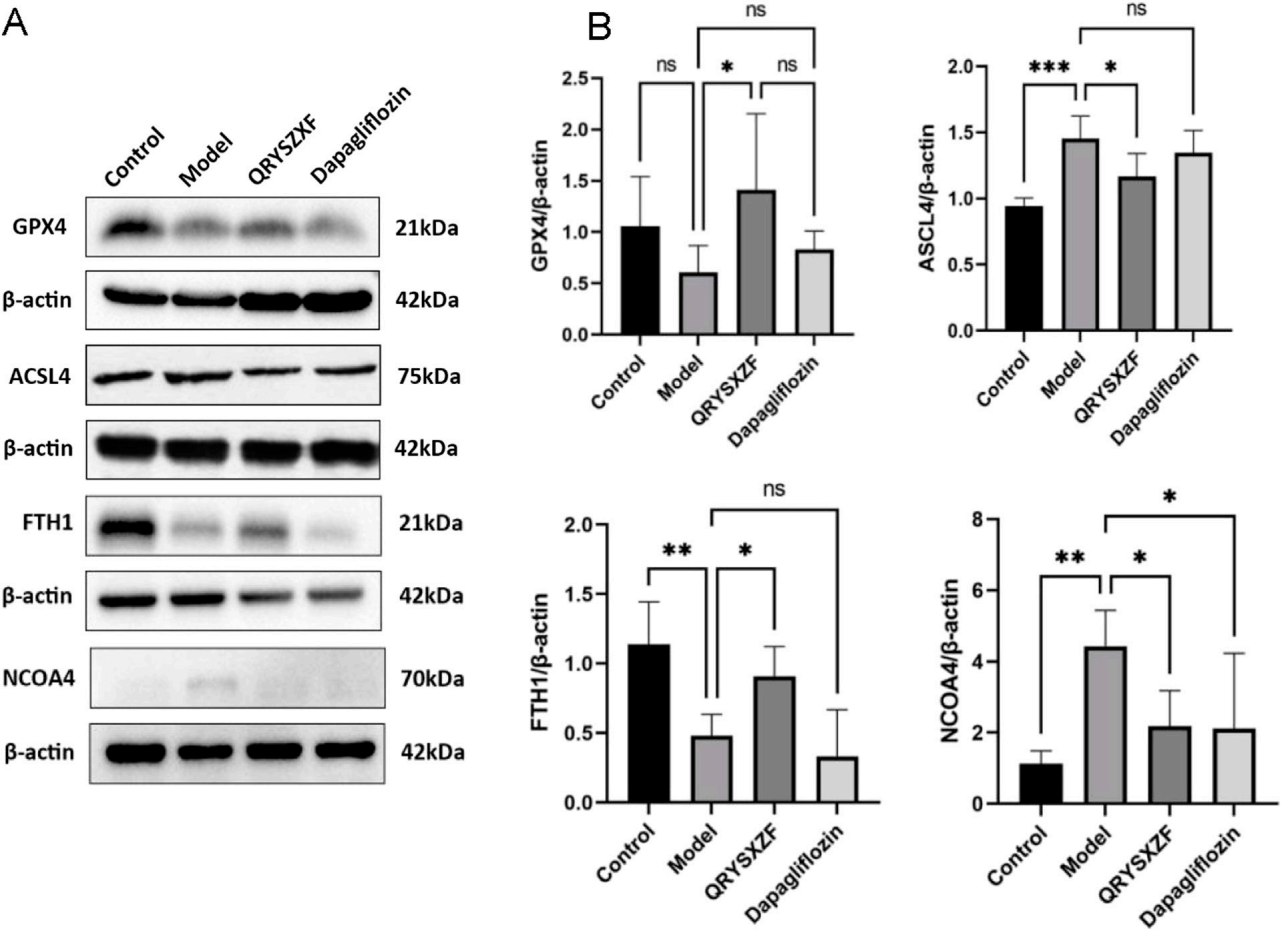
Comparative analysis of ferroptosis-related markers in renal tissues among experimental rat groups (n = 6). **(A)** Western blot analysis of GPX4, ACSL4, FTH1and NCOA4 protein expression in renal tissues across experimental rat groups. **(B)** The relative expression level of GPX4, ACSL4, FTH1and NCOA4 protein. *P < 0.05,**P < 0.01,***P < 0.001.

As shown by Western blot analysis (Figures 9). Compared with control group, the model group exhibited decreased renal expression of GPX4 (P > 0.05) and FTH1 (P < 0.01), along with significantly elevated ACSL4 and NCOA4 levels (P < 0.01). QRYSXZF treatment significantly upregulated GPX4 and FTH1 while downregulating ACSL4 and NCOA4 protein expression versus the model group (all P < 0.05). The dapagliflozin group showed no significant changes in GPX4, ACSL4 or FTH1 expression but demonstrated reduced NCOA4 levels (P < 0.05) compared to model controls.

### 3.10 QRYSXZF demonstrated significant inhibition of the HIF-1α/HO-1 signaling pathway

**FIGURE 10:**
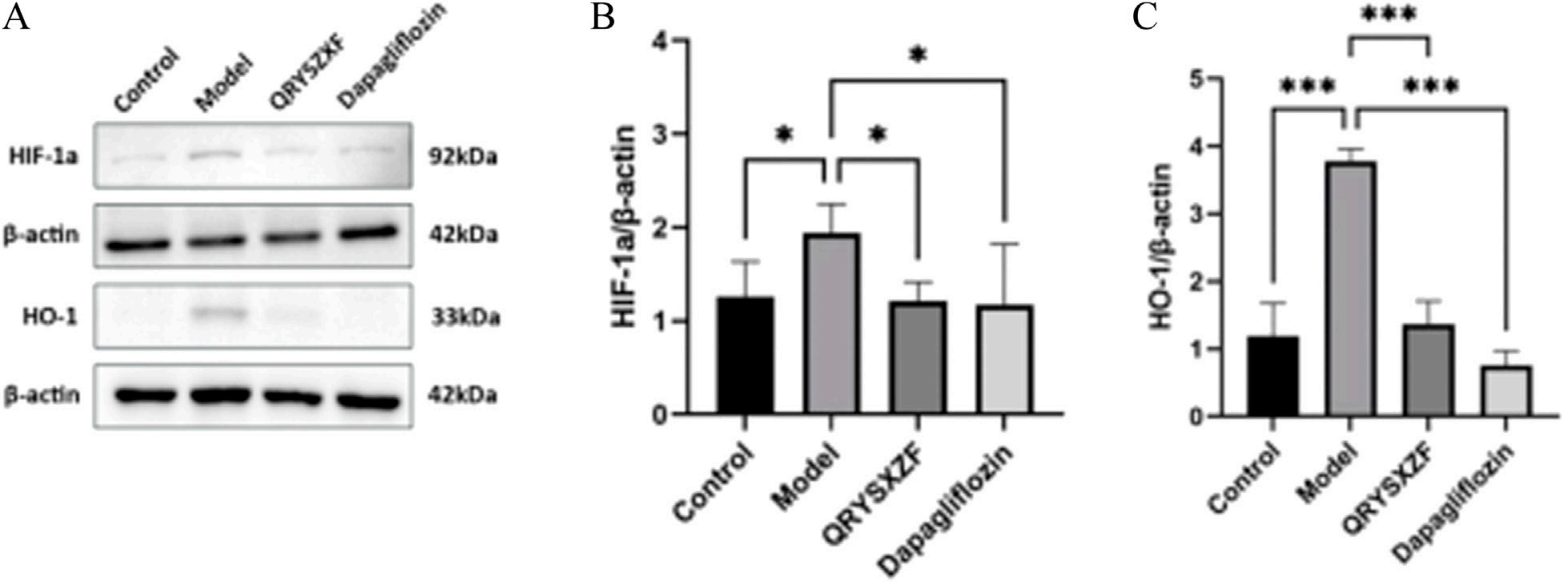
**(A)** Western blot analysis of HIF-1α and HO-1 protein expression in renal tissues across experimental rat groups (n = 6). **(B)** The relative expression level of HIF-1α protein (n = 6). **(C)** The relative expression level of HO-1 protein (n = 6). *P < 0.05,**P < 0.01,***P < 0.001.

As evidenced by Western blot analysis (Figure 10A–C) showing markedly elevated protein expression levels of both HIF-1α (*P* < 0.05) and HO-1 (*P* < 0.001) in renal tissues of the model group compared to the control group. Both QRYSXZF-treated and dapagliflozin-treated groups exhibited significantly reduced protein expression of HIF-1α (*P* < 0.05) and HO-1 (*P* < 0.001) relative to the model group.

## 4 Discussion

Network pharmacological analysis revealed that active components such as quercetin, kaempferol, β-sitosterol, luteolin, and isorhamnetin play pivotal roles in QRYSXZF’s therapeutic effects against DKD. Quercetin, a natural flavonoid compound, exerts renal protection by inhibiting oxidative stress, scavenging free radicals, modulating antioxidant pathways, reducing extracellular matrix accumulation, and suppressing renal tubular epithelial cell transdifferentiation (Gao et al., 2020). Additionally, it activates the Keap1/Nrf2/ARE signaling pathway to mitigate ferroptosis and preserve renal function (Wang et al., 2020b). Kaempferol demonstrates significant anti-inflammatory, antioxidant, and anti-tumor activities, with notable protective effects in DKD (Amjad et al., 2022). Studies indicate that kaempferol downregulates NADPH oxidase 4 (NOX4) expression to reduce ROS generation, thereby alleviating oxidative damage, while simultaneously upregulating HO-1 to ameliorate high glucose-induced oxidative stress and inflammation in renal tubular epithelial cells, inhibiting apoptosis and enhancing renal protection (Wang et al., 2024). β-Sitosterol improves glycemic control in type 2 diabetic rats by activating insulin resistance and glucose transporter 4 in adipose tissue, while also significantly lowering serum total cholesterol (TC) and triglycerides (TG) (Li et al., 2012), exhibiting potent antidiabetic and antioxidant properties (Gupta et al., 2011). Luteolin, a flavonoid with pro-autophagic and anti-inflammatory functions, offers multifaceted protection against diabetic renal injury. It modulates the renin-angiotensin system to ameliorate intraglomerular hypertension and prevent hyperglycemia-induced hemodynamic deterioration, while simultaneously regulating renal cell apoptosis and autophagy to suppress high glucose-induced excessive proliferation, thereby delaying DKD progression. Isorhamnetin has been shown to alleviate glomerular hypertrophy, basement membrane thickening, mesangial matrix expansion, and tubular epithelial cell damage in DKD rats, while enhancing SOD activity to improve oxidative stress and decelerate disease progression (Wang et al., 2021).

Using Cytoscape software, we constructed a protein-protein interaction (PPI) network and identified 16 core targets (including TNF, IL6, IL1β, ALB, AKT1, etc.), which are involved in multiple pathological processes such as inflammatory response, oxidative stress, apoptosis, and metabolic regulation. As key pro-inflammatory factors, TNF-α (Wang et al., 2018), IL-6, and IL-1β (Hirano, 2021) not only drive the formation of renal micro-inflammatory environment by activating NF-κB and MAPK signaling pathways, but also promote ROS generation. Together with iron overload, they synergistically induce lipid peroxidation, forming a vicious cycle of “inflammation-oxidative stress-ferroptosis”. Beyond serving as a biomarker, ALB exhibits antioxidant properties and maintains iron homeostasis, thereby alleviating renal tubular damage (Sally et al., 2022). AKT1 regulates autophagy and GPX4-mediated ferroptosis resistance through the PI3K/AKT/mTOR pathway, while HIF1A activates the TGF-β1/HO-1 pathway under hypoxic conditions, promoting iron accumulation and renal fibrosis (Manning and Toker, 2017). Additionally, PTGS2 (Ding et al., 2022), PPARG (Liu et al., 2010) and STAT3 (Zheng et al., 2019) participate in metabolic dysregulation, whereas CASP3, BCL2 and TP53 regulate apoptotic processes. The intricate interactions among these targets collectively constitute the complex pathological network of DKD.

Integrating the results of PPI network analysis, KEGG pathway enrichment, and literature review, we hypothesize that the HIF-1α/HO-1 pathway may serve as one of the key therapeutic targets of QRYSXZF in treating DKD. HIF-1, a crucial heterodimeric transcription factor composed of HIF-1α and HIF-1β subunits, plays a pivotal role in cellular responses to hypoxia by regulating the activity of HIF-1α subunit and modulating downstream target genes. It orchestrates various hypoxia-adaptive responses including angiogenesis, immune regulation, metabolic reprogramming, and apoptosis (Song et al., 2014). Studies demonstrate that early-stage DKD exhibits excessive HIF-1α activation due to microcirculatory dysfunction-induced ischemia and hypoxia (Askenazi et al., 2011), which promotes renal interstitial fibrosis, proteinuria, renal functional decline, and pathological morphological changes (Bessho et al., 2019). Under hypoxic conditions, suppressed hydroxylation of HIF-1α facilitates its dimerization with HIF-1β and subsequent nuclear translocation, activating hypoxia-responsive elements such as TGF-β1 and HO-1. This exacerbates oxidative stress-mediated ROS generation, enhanced lipid peroxidation, and iron accumulation in renal tubules, ultimately inducing ferroptosis and aggravating renal injury (Feng et al., 2021). HO-1, identified as a key downstream effector of HIF-1α in our study, demonstrated a high degree value (166) in the PPI network, indicating its central role. Furthermore, GO enrichment analysis revealed significant involvement in biological processes including “response to oxidative stress” and “positive regulation of oxidoreductase activity,” suggesting QRYSXZF may alleviate hyperglycemia-induced oxidative damage by restoring antioxidant defense systems. The “regulation of apoptotic signaling pathway” term encompassed multiple cell death modalities, including ferroptosis. Molecular docking results showed favorable binding affinities (binding energy < −4.25 kcal/mol) between HIF1A/HMOX1 and QRYSXZF’s active components (quercetin, kaempferol, β-sitosterol, luteolin, and isorhamnetin), with hydrogen bond interactions confirming their potential binding capabilities, thereby supporting the reliability of network pharmacology predictions.

Through network pharmacology prediction of QRYSXZF’s potential targets and pathways in DKD treatment, we established a DKD rat model to experimentally investigate its regulatory effects on the ferroptosis-related HIF-1α/HO-1 pathway and determine whether renal protection is achieved through ferroptosis inhibition. Compared with normal controls, model group rats exhibited significantly elevated blood glucose, kidney weight, 24h-UTP, and BUN levels. Histopathological analysis via HE, PAS, and Masson staining revealed glomerular hypertrophy, capillary loop/basement membrane/mesangial cell proliferation, and marked collagen deposition with fibrotic changes in diabetic kidneys. IHC confirmed upregulated expression of fibrotic proteins FN and TGF-β in model group kidneys, while both QRYSXZF and dapagliflozin interventions significantly ameliorated these pathological alterations, demonstrating their renal protective effects. Given the correlation between tubular dysfunction and renal fibrosis progression - where diabetic tubular injury is recognized as a fibrogenic driver - we evaluated tubular damage markers (Zhou et al., 2021). KIM-1 (a transmembrane protein specifically expressed in injured tubular epithelium) and NGAL (a kidney injury-associated secretory protein) showed significant elevation in model group, consistent with their established roles as independent predictors of DKD-to-ESRD progression and sensitive tubular injury indicators respectively (GENG et al., 2021). Both QRYSXZF and dapagliflozin effectively reduced these biomarkers’ expression. Furthermore, since DKD-induced proximal tubular apoptosis primarily occurs through caspase pathways (Aladaileh et al., 2021), we observed markedly increased Caspase-3 expression in model group rats, which was significantly attenuated by QRYSXZF treatment, indicating its anti-apoptotic effect on diabetic renal tubules.

Oxidative stress serves as a critical factor in ferroptosis pathogenesis, where chronic hyperglycemia in DKD activates oxidase systems to generate excessive ROS that overwhelms endogenous antioxidant capacity, leading to oxidative stress and lipid peroxidation (Fang et al., 2023). Key biomarkers of this process include the lipid peroxidation byproduct MDA and enzymatic antioxidants such as SOD, CAT, and GSH-Px. GPX4 plays a pivotal role in regulating ferroptosis by reducing lipid peroxides; its impaired activity diminishes redox capacity, allowing Fe^2+^-mediated Fenton reactions to propagate lipid oxidation and ROS accumulation, ultimately triggering ferroptosis (Kuang et al., 2020). Our results demonstrated elevated MDA and decreased SOD/GSH-Px levels in DKD rat kidneys compared to normal controls, while QRYSXZF treatment effectively reversed these alterations and upregulated GPX4 expression, indicating its ability to counteract lipid peroxidation-driven ferroptosis in DKD progression. Another crucial ferroptosis mechanism involves NCOA4-mediated ferritinophagy, where ferritin (the primary intracellular iron storage protein) degradation releases iron to fuel Fenton reactions. Studies show that genetic silencing of NCOA4 or pharmacological inhibition of NCOA4-FTH1 interaction stabilizes FTH1, limits Fe^2+^ release, and confers ferroptosis resistance (Mancias et al., 2014). Our data revealed increased SI and SF alongside decreased TF in DKD model rats, with QRYSXZF treatment significantly lowering SI/SF while slightly elevating TF. Western blot analyses confirmed upregulated NCOA4 and downregulated FTH1 in model group kidneys, demonstrating NCOA4-mediated ferritin degradation and subsequent iron overload in DKD, which QRYSXZF effectively ameliorated by reducing NCOA4 expression and preserving FTH1, thereby attenuating ferroptosis. These findings collectively suggest that QRYSXZF alleviates DKD by dual mechanisms: restoring redox homeostasis through GPX4 activation and modulating iron metabolism via NCOA4/FTH1 regulation.

The pathogenesis of DKD is intrinsically linked to renal hypoxia, a well-established phenomenon resulting from the kidney’s unique physiological demands and exacerbated by diabetes-induced vascular dysfunction. Renal tubules, owing to their intense reabsorptive workload, exhibit extraordinary oxygen consumption rates that render them exquisitely sensitive to ischemic insults. This vulnerability is amplified in the diabetic milieu, where chronic hyperglycemia, metabolic derangements, and microcirculatory impairment converge to create a perfect storm of tubular hypoxia and subsequent injury, driving DKD progression (Feng et al., 2021). At the molecular level, the cellular response to hypoxia is masterfully orchestrated by HIF-1α, which stabilizes under low oxygen tension and transcriptionally activates a battery of adaptive genes. Among these, HO-1 emerges as a particularly consequential effector, forming with HIF-1α a pivotal axis in the renal hypoxia response network. While transient activation of this pathway confers cytoprotection through metabolic flexibility and antioxidant defense mechanisms, its chronic induction paradoxically contributes to renal pathology through multiple well-characterized mechanisms (Mancias et al., 2014). Sustained HIF-1α signaling has been conclusively shown to promote extracellular matrix deposition (Yan et al., 2024) and fibrotic transformation in diabetic kidneys (Wang and Ye, 2019), whereas HO-1 overexpression drives iron dysregulation through accelerated heme catabolism, creating a permissive environment for ferroptosis via iron-catalyzed lipid peroxidation cascades (Clara et al., 2020). Our experimental findings demonstrate that QRYSXZF treatment effectively normalizes the pathological overexpression of both HIF-1α and HO-1 in DKD kidneys, while concurrently ameliorating established markers of ferroptosis and iron overload. These observations, when considered alongside emerging evidence that HO-1-mediated iron handling directly influences tubular epithelial cell fate in diabetes (Clara et al., 2020), strongly support our hypothesis that QRYSXZF exerts its renoprotective effects at least partially through modulation of the HIF-1α/HO-1 axis. Mechanistically, this may involve: (i) improvement of medullary oxygenation through microvascular protection, thereby reducing HIF-1α stabilization; (ii) direct interference with HIF-1α protein degradation or DNA-binding activity; and (iii) normalization of HO-1-dependent iron recycling pathways to prevent cytotoxic iron accumulation. The convergence of these actions would collectively restore redox homeostasis and mitigate ferroptotic stress in vulnerable tubular compartments. Future studies employing cell-specific knockout models and isolated bioactive compounds will be instrumental in delineating the precise molecular interactions underlying these therapeutic effects.

## 5 Conclusion

This study, through network pharmacological analysis and animal experimentation, demonstrates that QRYSXZF may alleviate renal function impairment and renal fibrosis while delaying DKD progression by inhibiting the HIF-1α/HO-1 pathway, thereby reducing iron accumulation and oxidative stress to mitigate ferroptosis. These findings contribute to further understanding of DKD pathogenesis and provide novel therapeutic targets for DKD treatment.

However, the following limitations remain: The specific active components of QRYSXZF and their *in vivo* metabolic pathways require further isolation and identification; the necessity of the HIF-1α/HO-1 pathway has not been validated using gene knockout models. Future studies will combine organoid culture and single-cell sequencing to deepen the exploration of the underlying mechanisms.

## Data Availability

The original contributions presented in the study are included in the article/supplementary material, further inquiries can be directed to the corresponding authors.
